# Proteomic Analysis of Anticancer TCMs Targeted at Mitochondria

**DOI:** 10.1155/2015/539260

**Published:** 2015-10-19

**Authors:** Yang Wang, Ru-Yuan Yu, Qing-Yu He

**Affiliations:** Key Laboratory of Functional Protein Research of Guangdong Higher Education Institutes, Institute of Life and Health Engineering, College of Life Science and Technology, Jinan University, Guangzhou 510632, China

## Abstract

Traditional Chinese medicine (TCM) is a rich resource of anticancer drugs. Increasing bioactive natural compounds extracted from TCMs are known to exert significant antitumor effects, but the action mechanisms of TCMs are far from clear. Proteomics, a powerful platform to comprehensively profile drug-regulated proteins, has been widely applied to the mechanistic investigation of TCMs and the identification of drug targets. In this paper, we discuss several bioactive TCM products including terpenoids, flavonoids, and glycosides that were extensively investigated by proteomics to illustrate their antitumor mechanisms in various cancers. Interestingly, many of these natural compounds isolated from TCMs mostly exert their tumor-suppressing functions by specifically targeting mitochondria in cancer cells. These TCM components induce the loss of mitochondrial membrane potential, the release of cytochrome c, and the accumulation of ROS, initiating apoptosis cascade signaling. Proteomics provides systematic views that help to understand the molecular mechanisms of the TCM in tumor cells; it bears the inherent limitations in uncovering the drug-protein interactions, however. Subcellular fractionation may be coupled with proteomics to capture and identify target proteins in mitochondria-enriched lysates. Furthermore, translating mRNA analysis, a new technology profiling the drug-regulated genes in translatome level, may be integrated into the systematic investigation, revealing global information valuable for understanding the action mechanism of TCMs.

## 1. Introduction

Traditional Chinese medicine (TCM) has been used for thousand years in China. There is a well-established theoretical approach in TCM treatment based on Chinese philosophy. According to Chinese medicine, diseases resulted from a disturbance of the balance that maintains health (yin-yang balance). Physicians adopt different combinational formulas of TCM to regulate the harmony of the body-mind-environment network of patients according to the syndromes, age, gender, and physique [1], and therefore patients in different backgrounds receive specific treatments, equivalent to modern medical conception-personalized therapy.

However, for a long time TCM had been treated with skepticism in academic medicine because of the lack of herbal standardization and quality control [2] and the ambiguity of functional molecules and their action mechanisms. Beginning from past decades, increasing studies with modern chemical, biochemical, and molecular biological methods showed that TCMs are rich with various functional compounds active in cancer therapy [3–6]. There is a revival of interest in TCMs and many scientists turn to explore the action mechanisms of the bioactive natural products in cellular and molecular levels.

The mainstream strategy to study TCMs is to isolate and purify bioactive components from herbs or animals, observe their biological and medical effects in cellular and animal models, and then investigate the signaling pathways involved by the compounds in molecular level [7]. Up to now, thousands of active components have been isolated from TCMs and their potentials for the treatment of cancer, cardiovascular disease, and diabetes have been explored. However, the technologies for holistically investigating and understanding the mechanisms of TCMs are limited. Systems biology is regarded as the possible method that can bring breakthroughs in the study of TCM [8], because its advantage is in accord with the holistic philosophy of Chinese medicine. Based on the systems theory, multiomics strategies [9] and multiple-target approaches must be the good choices for molecular screening, providing global views for elucidating the essence and molecular basis of TCMs. Meeting the urgent need for the high-throughput technologies, proteomics, as a powerful tool of systems biology, can be used to profile the differential expression of proteins in response to the biological action by TCM compounds, summarizing the top molecular pathways induced by the compounds and then the complex mechanisms can be further investigated in detail [10].

## 2. TCMs Induce Cancer Cell Death in Mitochondrial-Dependent Pathway

**Figure 1:**
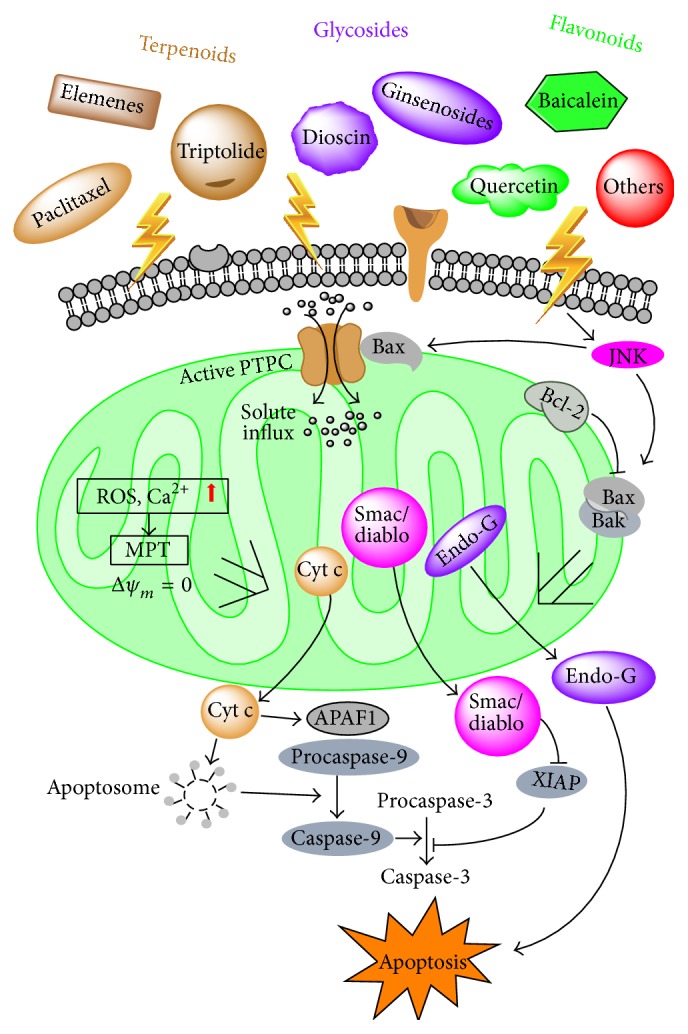
Modulation of mitochondrial-dependent apoptosis pathways by natural compounds. Mitochondrial transmembrane potential (Δ*ψ*_*m*_) is maintained by permeability transition pore complex (PTPC). PTPC interacts with Bcl-2, contributing to the exchange of small metabolites between the cytosol and the mitochondrial matrix. When the cell suffers stimuli from natural compounds, the accumulation of reactive oxygen species (ROS) and Ca^2+^ activates the PTPC, favored by its interaction with Bax, allowing small solutes to get into the mitochondrial matrix, resulting in decreased Δ*ψ*_*m*_. This eventually leads to mitochondrial outer membrane permeabilization, where many cytotoxic proteins such as cytochrome c, diablo, and endonuclease G are released to cytoplasm, initiating apoptotic signaling.

Mitochondrion is the key regulator in cellular energy homeostasis and plays a central role in determining cell apoptotic process [11, 12]; it is therefore regarded as a vital target for cancer chemotherapy [13]. Many investigations revealed that bioactive compounds can act on mitochondria to trigger the permeabilization of the mitochondrial outer membrane and lead to the impairment of the mitochondria, including the alteration of electron transport, the loss of mitochondrial transmembrane potential, and the cytosolic release of apoptotic proteins such as cytochrome c (Figure 1). Our previous studies based on proteomics also demonstrated that many natural active molecules, including* isodeoxyelephantopin* [14],* andrographolide analogue* [15, 16],* tubeimoside-1* [17], and* dioscin* [18] extracted from TCMs, induce cancer cell apoptosis mainly in mitochondria-dependent pathway. Mitochondria are likely the primary and common targets for TCM compounds as suggested by proteomic profiling, showing the substantial TCM-induced alterations of mitochondrial proteins among others. In this paper, we attempt to discuss the functional roles of several TCM compounds with anticancer properties, with special emphasis on the involved molecular mechanism* via* mitochondria as cellular targets using proteomics as a primary screening technology.

### 2.1. Terpenoids

**Figure 2:**
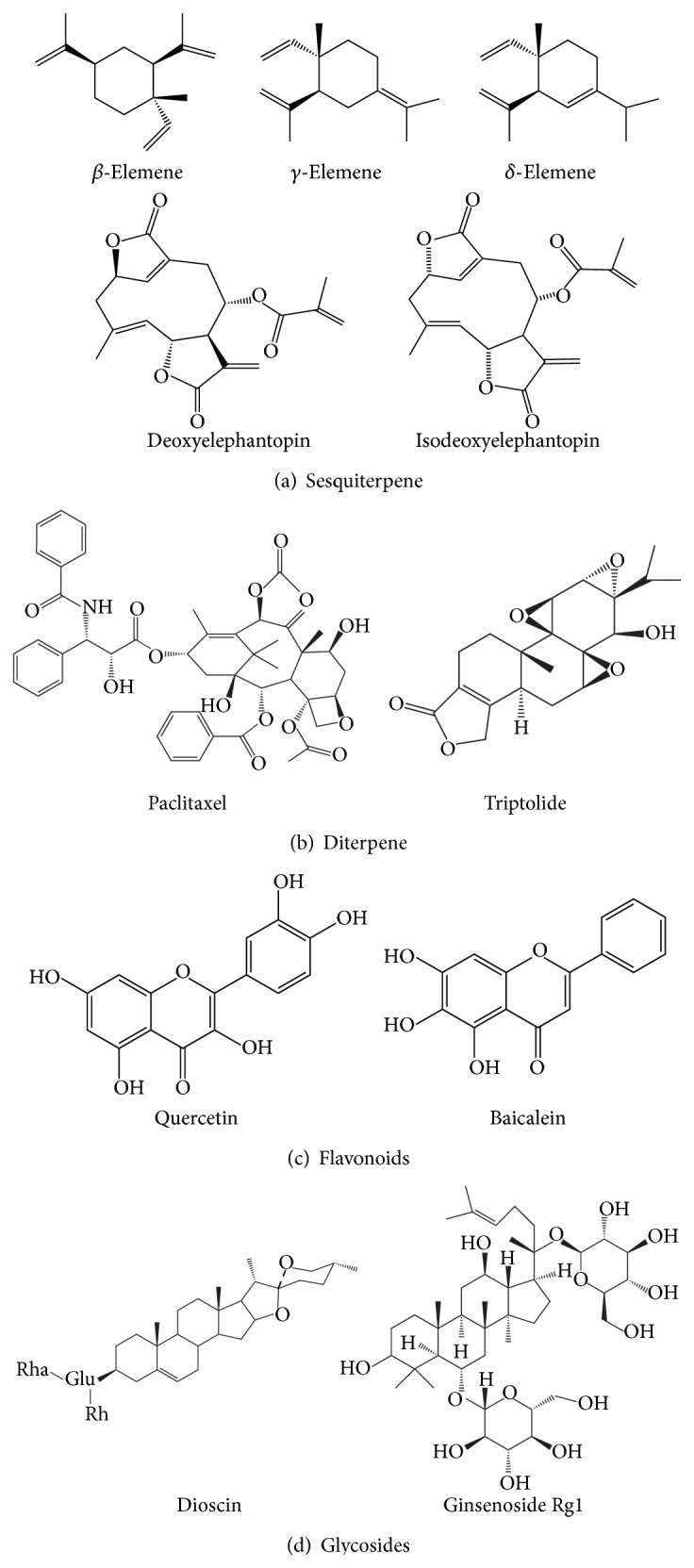
Chemical structures of compounds isolated from TCMs.

Terpenoids are the largest and diverse class of natural products, which can be found in all classes of living things. These compounds feature five-carbon isoprene units assembled and modified in thousands of ways. Figures 2(a) and 2(b) display several structures of terpenoids extracted from TCM herbs. Accumulating reports demonstrated that many terpenoids exhibit strong effects on preventing carcinomas, as shown in Table 1.

#### 2.1.1. Effects of Sesquiterpene on Mitochondria

Elemene is a sesquiterpene extracted from the TCM herb* Curcuma wenyujin* and* Curcuma zedoaria* Roscoe [19], including *β*-elemene, *γ*-elemene, and *δ*-elemene (Figure 2(a)). Among them, *β*-elemene has been widely used to inhibit cancer. A study demonstrated that *β*-elemene is able to reverse the drug resistance of A549 cells by decreasing the mitochondrial membrane potential, in which the membrane damage initiates apoptosis process* via* cytochrome c release, caspase activation, and the modulation of the expression of Bcl-2 family proteins [20]. Moreover, *β*-elemene can augment the cisplatin activity and carry out a synergistic effect on disrupting the mitochondrial transmembrane potential, inducing apoptosis in ovarian carcinoma cells [21]. Through targeting mitochondria, the antitumor effect of *β*-elemene was also observed in prostate, brain, breast, cervical, and colon cancers [22]. Research with iTRAQ-based proteomics revealed that several pathways in gastric cancer (SGC7901) may be involved by *β*-elemene, including ribosome signaling, peroxisome proliferator-activated receptors (PPARs) signaling, regulation of actin cytoskeleton, phagosome, biosynthesis, and metabolism of amino acids [23]. In particular, as observed by the proteomic study, the expression of p21-activated protein kinase-interacting protein 1 (PAK1IP1) and Bcl-2-associated transcription factor 1 (BTF) is significantly regulated by *β*-elemene, indicating the antitumor effect probably by targeting mitochondria, though the binding proteins remain to be explored.

Deoxyelephantopin (ESD) and isodeoxyelephantopin (ESI) are two germacranolide sesquiterpene lactones (Figure 2(a)) isolated from TCM herb* Elephantopus scaber* [24–26]. This herb is a folk medicine usually used for preventing and treating respiratory diseases in China. However, accumulating evidences demonstrated that both ESD and ESI are able to induce cell apoptosis in TS/A cells (lung cancer) and CNE1 (nasopharyngeal carcinoma). Their underlying molecular mechanisms were further characterized with the advanced proteomic technologies. Lee et al. used 2DE DIGE and LC-ESI-MS/MS to profile proteins that are significantly regulated by ESD treatment in TS/A cells [27]. As revealed in this experiment, protein alternations regulated by ESD are involved in proteolysis and calcium ion transport, indicating that ESD may target proteasome and endoplasmic reticulum in TS/A cells. At the same time, Su and colleagues found that ESD is able to inhibit the cell proliferation, induce cell cycle arrest, and trigger apoptosis in CNE cells by decreasing the mitochondrial membrane potential (Δ*ψ*_*m*_) [25]. SILAC (stable isotope labeling with amino acids in cell culture) quantitative proteomics coupled with bioinformatics was also made full use of to characterize the molecular mechanism of ESI in nasopharyngeal carcinoma [14]. ESI was found to provoke G2/M arrest and apoptosis by inducing ROS generation, in which the accumulated ROS promote DNA breakage and mitochondrial-mediated apoptosis. Obviously, mitochondria are likely a key target, among others, for sesquiterpene.

#### 2.1.2. Effects of Diterpene on Mitochondria

Paclitaxel (taxol), a famous diterpene plant product extracted from the* Taxus brevifolia* in 1971, has been currently employed as an antimitotic agent in chemotherapy for the treatment of various human cancers. The structure is showed in Figure 2(b). It is well known that taxol can induce cell apoptosis by preventing tubulin depolymerization during mitosis [28, 29], particularly in lung cancer and ovarian cancer [30, 31]. Mounting clinical evidences proved that many cancers acquire paclitaxel resistance during chemotherapy because of heterogeneity. Thereby growing researches, based on proteomics, aimed to reveal the complex molecular mechanisms of paclitaxel resistance in cancers [31–35].  Table 3 lists several paclitaxel resistance studies determined by proteomics in different cancers.

In lung cancer, a prevalent research strategy is to establish paclitaxel-resistant tumor subline and parental-sensitive cell line* via* stepwise selection by paclitaxel and then compare the differentially expressed proteins in two cell lines by proteomics. With such a method, Sun's group used 2DE DIGE to identify 30 altered proteins, which mainly belong to signal transduction, cytoskeleton, redox reaction, energy, and metabolism [35]. Another proteomic study found that the treatment of paclitaxel combined with MEK inhibitor significantly alters the level of RS/DJ-1 (RNA-binding regulatory subunit/DJ-1 PARK7) and RhoGDI*α* (Rho GDP-dissociation inhibitor *α*) in NSCLC H157 cell line, suggesting an important role that RS/DJ-1 and RhoGDI*α* are involved in drug resistance [30]. Furthermore, Tian and his colleagues employed multiple quantitative proteomic methods (iTRAQ labeling and label-free) to analyze paclitaxel resistance associated proteins in ovarian serous carcinoma cell line (SKOV-3) [31]. This in-depth proteomic screening identified 1371 differential proteins, including mitochondrial trifunctional enzyme, mitochondrial ATP synthase, complement component 1 Q subcomponent binding protein, cytochrome c, GrpE protein homolog 1, mitochondrial inner membrane protein, and mitochondrial malate dehydrogenase, suggesting that mitochondria play a core role in responding to paclitaxel treatment. These observations indicate that advanced proteomic techniques should be applied to obtain comprehensive views on protein alterations, in which mitochondria associated pathways can be fairly evaluated.

Triptolide, also called* Tripterygium wilfordii lactone alcohol*, is an oxygenated diterpene isolated from the Chinese herb* Tripterygium wilfordii* HOOK F, which contains three epoxy groups of diterpene lactone compounds (Figure 2(b)). Present studies showed that triptolide possesses high toxicity and thus can exert proapoptotic and antiproliferative effects on multiple tumor cell lines* in vitro* [36–38]. Comprehensive proteomics was employed to determine the triptolide-regulated proteins, which are related to oxidative stress, mitochondria, and signal transduction, confirming that triptolide inhibits the activation of JNK and p38 by decreasing ROS level [39]. The toxic effect of triptolide in normal liver cell (L-O2) was also investigated; the observed loss of mitochondrial membrane potential and cytochrome c releasing suggests that triptolide can also work on the mitochondria in normal cells to induce apoptosis [40].

### 2.2. Flavonoids

Flavonoids are kinds of natural products with abundant contents in TCM herbs, fruits, and vegetables. They are a group of polyphenolic compounds containing more than 6000 flavonoids, which are divided into 6 subclasses, including flavonols, flavanols, isoflavones, anthocyanidins, flavanones, and flavones [41]. Some epidemiological studies reported that intake of flavonoids may be uncertain or even harmful to cancer therapy [42, 43]; nevertheless, the majority of evidences supported their potential cancer protective properties [44–46]. Regarding this controversial role of flavonoids in cancer, proteomics may provide a global picture with comprehensive information for assessment. As shown in Table 2, the action mechanisms of some well-known flavonoids have been characterized by proteomics.

Quercetin (3,3′,4′,5,7-pentahydroxyflavone) is a major flavonoid compound in fruits, which possesses a wide range of biological activities, including antioxidant [47], antitumor [48], and metabolic regulation [49] (Figure 2(c)). A proteomic study using SILAC method found that quercetin inhibits HepG2 proliferation and migration by regulating IQGAP1 and *β*-tubulin expression [50]. Experimental results showed that quercetin-regulated proteins are involved in multipathways, including antioxidation-relating pathway and mitochondria-dependent apoptosis pathway. To understand the relationship between high fruit intake and the risk of colon cancer, Mouat et al. used 2DE-based proteomics to determine the influence of quercetin on human colon adenocarcinoma cell line (SW480), revealing that type II cytoskeletal 8 keratin and NADH dehydrogenase (ubiquinone) Fe-S protein 3 are downregulated, while the annexin family related that proteins are upregulated [51]. These data suggest that quercetin may decrease tumorigenicity through impairing the transfer of electrons from NADH to ubiquinone in the respiratory chain; that is, mitochondria may be the potential target of quercetin. With regard to metabolic regulation, a study applied transcriptome and proteome profiling to investigate the effect of quercetin on colon mucosa in rats model, indicating that mitogen-activated protein kinase (MAPK) pathway is downregulated while phases I and II metabolism pathway, PPAR*α*, and mitochondrial fatty acid degradation pathway are enhanced [52]. This observation implicates that quercetin may induce a shift in energy production pathways from decreased cytoplasmic glycolysis to increased mitochondrial fatty acid degradation during cancer development.

Baicalein, the main active component isolated from* Scutellaria baicalensis*, is a flavonoid that shows cytotoxic effect on various human cancer cell lines [53, 54] and also plays a vital role in protecting cell against surrounding stress [55] (Figure 2(c)). To better illustrate the antitumor effect of baicalein on colorectal cancer, Huang et al. used 2DE-proteomic approach to identify 11 differentially expressed proteins as the potential targets of baicalein [56]. Peroxiredoxin-6 (PRDX6), an upregulated protein after baicalein treatment, was found to decrease the generation of ROS and inhibit the growth of colorectal cancer cells. Similarly, antiproliferation effect of baicalein was also reported in bladder cancer T24 cells, showing that baicalein inhibits Akt signaling and downregulates Bcl-2 expression [57]. At the same time, loss of mitochondrial membrane potential and activation of caspase-9 and caspase-3 were observed, implicating that baicalein induces apoptosis* via* mitochondrial-dependent caspase activation pathway. Nevertheless, an opposing observation was also reported: a baicalein isolated from* Scutellaria baicalensis* extract (SbE) increases colorectal cancer cell growth, whereas SbE without baicalein significantly induces mitochondrial apoptotic pathway [58]. This discrepancy remains to be reassessed by using advanced systems biological technologies including proteomics.

### 2.3. Glycosides

Glycosides are another class of natural products widely stored in living organisms. They can transform into active status through enzyme hydrolysis with their sugar groups broken off. These sugar groups of glycosides usually and susceptibly interact with toxic compounds from surroundings and are easily eliminated from the body. Recently, with the application of proteomics, increasing studies focused on the anticancer effects of glycosides. Here are some examples as shown in Table 2.

Dioscin (Figure 2(d)), a typical glucoside saponin derived from TCM plants* Polygonatum zanlanscianense* pamp, shows multiple pharmacological activities such as apoptosis induction in various carcinoma cell lines [59, 60] and liver injury protection [61]. Regarding the target of dioscin, accumulating evidences pointed to mitochondria. Earlier work in our laboratory used 2DE-based proteomics to profile the proteomic changes in response to dioscin treatment in human myeloblast leukemia HL-60 cells, revealing that dioscin exerts cytotoxicity* via* mitochondrial apoptotic pathway [18]. Our further investigation demonstrated that dioscin is capable of inducing mitochondria dysfunction and reactive oxygen species (ROS) generation with decreased Δ*ψ*_*m*_, leading to the initiation of the death receptor signaling pathway.

In parallel, ROS generation was observed as well in human colon cancer cells (HCT-116) after dioscin stimulation. Peng's laboratory employed iTRAQ-based proteomics to analyze the cytotoxic mechanism of dioscin in HCT-116 cells and identified 288 differentially expressed proteins, which are involved in oxidative phosphorylation, Wnt, p53, and calcium signaling pathways [62]. By regulating mitochondria, dioscin exerts not only proapoptotic effect but also hepatoprotective function in acetaminophen-induced liver injury [61]. As screened by 2DE-proteomics, 15 dioscin-regulated proteins probably associated with hepatoprotection are identified, including Suox, Krt18, Rgn, Prdx1, MDH, and PNP. In addition, dioscin is able to mediate Ca^2+^ balance* via* regulating Rgn and upregulating Ktr18 in cells that suffer from acetaminophen-induced mitochondrial damage.

Ginseng (*Panax ginseng* Meyer) is a medicinal herb of the family Araliaceae; its root has been commonly used for keeping healthy in China over 2000 years [63]. Ginsenosides, the major active compounds of ginseng, were reported to possess anticancer [64, 65], antimutagenic, anti-inflammatory, antidiabetes, and neurovascular effects [66]. Ginsenoside Rg1 is one of the ginsenosides that belong to triterpene glycosides (Figure 2(d)). It was reported to reverse TNF-*α*-attenuated nitric oxide production in human umbilical vein endothelial cells by a proteomic-based study [67]. TNF-*α* stimulation increases the expression of MEKK-3, reticulocalbin, phosphoglycerate, zinc finger protein, NSAP1 protein, and 6-phosphogluconolactonase, with reduced nitric oxide synthase. However, all these alterations can be restored by ginsenoside Rg1 pretreatment, suggesting a protective role of ginsenoside Rg1 in alleviating the injury of inflammatory factor on vascular disease.

The protective effect of ginsenoside Rg1 can be observed as well in cardiomyocytes [68]. Hypoxia condition induces neonatal rat cardiomyocytes death in mitochondrial apoptotic pathway, including ROS accumulation, loss of mitochondrial membrane potential, and cytochrome c release; nevertheless, ginsenoside Rb1 can markedly inhibit this process. Mitochondria are the arbiter in cardiomyocytes injuries by releasing apoptogenic proteins into the cytosol [69]; however, the exact targets of ginsenoside Rb1, probably associated with mitochondria, remained to be investigated.

### 2.4. Others

Honokiol (HNK) is a neolignan isolated from TCM herb* Magnolia officinalis* (also named* Houpu* in Chinese), exhibiting various pharmacological effects in preclinical experimental models [70]. A large volume of evidences demonstrated that HNK is able to induce apoptosis and antiproliferation in several cancers [71] and thus is regarded as a promising chemotherapy candidate in cancer therapy. In acute myeloid leukemia, HNK shows antileukemia effect by inhibiting enzyme activity of histone deacetylases, followed by the upregulation of p21/waf1 and Bax, leading to apoptosis [72]. HNK was also reported to promote cytoprotective autophagy mediated by ROS signaling in prostate cancer cells [73]. Using quantitative proteomic method (SILAC), Liang and colleagues found that HNK treatment in HepG2 is able to modulate cell migration by the downregulation of Ras GTPase-activating-like protein (IQGAP1), which interacts with Cdc42/Rac1 [74]. The interaction links to VEGFR-2/3 pathway to reduce cancer metastasis and proliferation. Another proteomic-based study profiled HNK-regulated proteins in Hela cells, showing 8 proteins with upregulation and 77 proteins with downregulation [75]. GO analysis revealed that 10% of these proteins are located in mitochondria, melanosome, and lysosome and over 17% are associated with metabolism, suggesting that HNK induces cell apoptosis* via* mitochondria signaling pathway, further confirming the previous result from Yang's group [76].

## 3. The Role of TCM-Regulated Proteins in Cancer Therapy

With the progresses in TCM investigation by proteomics approach, increasing TCM-regulated proteins were determined. Many TCMs were observed to suppress tumor development by regulating oncogenes. Can these TCM-regulated proteins serve as biomarkers to monitor cancer progression and measure treatment effectiveness?

Heat shock proteins (HSPs) are stress-inducible proteins including HSP100, HSP90, HSP70, HSP60, HSP40, and small HSPs; they act as molecular chaperones to regulate protein folding and transport, allowing cells to survive in lethal environments. In cancer, cells with higher metabolic requirement need more chaperones to maintain survival; these proteins are strong antiapoptotic proteins [77] and thus are regarded as antitumor targets in cancer therapy. Increasing reports revealed that many TCMs induce cancer cells apoptosis by decreasing HSPs [78–80]. Tanshinone IIA, an active component extracted from the roots of* Salvia miltiorrhiza* Bunge, was shown to inhibit HSP27 and promote apoptosis in cervical cancer [79]. Bufalin, a primary active ingredient of TCM* Chan-Su*, has the capacity of downregulating p-AKT and HSP27 and activating procaspase-3, procaspase-9, finally leading to cell apoptosis in pancreatic cancer [78]. HSPs, especially HSP90, are now confirmed to be essential for malignant transformation and progression [81]. In clinic, HSPs inhibition by TCMs might make the cancer cells more sensitive to chemotherapy.

NF-*κ*B signaling is the well-known pathway that mediates immune and inflammatory responses in cells. The family includes p65 (RelA), NF-*κ*B1 (p105/p50), and NF-*κ*B2 (p100/p52), able to dimerize in numerous combinations and determine the fate of cells. NF-*κ*B is constitutively activated and correlated with increasing grades in many tumors; it is thus received as the essential target for cancer therapy. To our knowledge, a great number of TCMs are able to suppress NF-*κ*B and induce apoptosis. Isodeoxyelephantopin isolated from* E. scaber* [82], ginsenosides isolated from* ginseng* [64, 65], and isorhamnetin isolated from pollen* Typha angustifolia* or* Hippophae rhamnoides* L. [83] have the capacity of inhibiting NF-*κ*B and exerting anti-inflammatory effect in cancers. In clinic, the subcellular localization of NF-*κ*Bs determined by immunohistochemistry is the convictive biomarker for monitoring cancer progression.

Drug resistance is emerging as a big challenge in cancer therapy and mitochondria play a vital role in this progression. Bcl-2 family is a group of mitochondrial proteins that regulate a diverse range of death signals. Among them, Bcl-2 is a strong antiapoptotic protein; its overexpression results in a more aggressive and treatment resistant phenotype [84]. A meta-analysis showed that Bcl-2 expression is a poor prognostic marker in lung cancer [85] and can be used as drug resistant marker as well. Some natural products, *β*-elemene extracted from TCM herb* Curcuma wenyujin* and triptolide isolated from* Tripterygium wilfordii* HOOK F, are able to inhibit Bcl-2 expression and thus sensitize cancer cells to chemotherapy [20, 86]. Besides, mitochondrial malate dehydrogenase also plays an essential role in docetaxel resistance in clinic [31, 87]; knockdown of malate dehydrogenase 2 (MDH2) increases docetaxel sensitivity by inducing metabolic inefficiency. Taken together, mitochondrial proteins are the arbitrator on drug resistance; therefore, the combination of mitochondrial protein inhibitors with chemotherapeutics may be the efficient method in cancer therapy.

## 4. The Application of Proteomics in TCMs in Cancer

**Figure 3:**
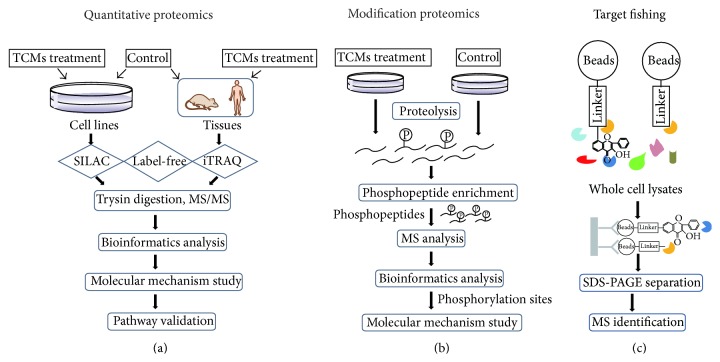
Scheme of approaches for the application of proteomics in the mechanistic study of TCMs. (a) Overview of conventional quantitative proteomics on TCMs including SILAC, label-free, and iTRAQ. Among them, SILAC is suitable for cell models, while label-free and iTRAQ can be applied to samples from various sources. Samples can be analyzed by MS after trypsin digestion. After comparison, the differently expressed proteins are sorted out by bioinformatics and selected for further mechanism studies. (b) The procedure of modification proteomics on TCMs investigation, with phosphoproteomics as a widely used approach. Samples are digested into peptides, followed by enrichment and MS analysis. (c) Abridged general view of target fishing. Beads linked with certain components of TCMs are used to capture “preys” in whole cell lysates; the target proteins can then be identified by mass spectrometry.

Proteomics is a multifunctional tool in investigating the mechanisms of TCMs, not only providing global views of molecular alterations induced by TCMs but also identifying protein-drug interactions. Quantitative proteomics such as SILAC and iTRAQ provides conventional ways to study the mechanism of TCMs. As shown in Figure 3(a), samples including cell lines or tissues with and without TCM treatments are labeled with various tags; the cell lysates are then digested by trypsin, followed by MS analysis. The proteins with expression alterations can be further analyzed by bioinformatics to uncover the signaling pathways regulated by TCMs.

In addition, posttranslational modifications are the most common phenomenon in eukaryotic cells; many proteins are activated or inactivated after posttranslational modifications including phosphorylation, ubiquitination, and glycosylation. The rising modification proteomics provides efficient methods to investigate the action of TCMs.  Figure 3(b) shows the whole procedure of phosphoproteomics: proteins from different TCM treatments are digested by trypsin, and the phosphopeptides are enriched by TiO_2_ and then analyzed by LC/MS. The global identification of modification sites offers us new insights into the action mechanism of TCMs.

For the target identification, magnetic “Fishing” assay [88] coupled with proteomics is an effective approach to screen protein-drug interactions, as shown in Figure 3(c). Single agents of TCMs are linked to magnetic beads, with affinity-based isolation; the target proteins that interact with the agents of interest can be identified by mass spectrometry; bioinformatics analysis such as molecular docking can further calculate the binding sites of TCMs.

It must be noted that although TCMs are purified as single agents, they may have more than one or multiple targets in tumor cells and their actions may be involved in more than one or several pathways. Proteomics coupled with bioinformatics is able to identify and integrate various signaling pathways of each agent thus figuring out which component is toxic to human body. Removing the useless components and optimizing the prescription of TCMs by proteomics can provide directive effects for developing therapeutic regimens.

## 5. The Development of Proteomics in Studying TCMs

With the rapid development of technology, proteomics progresses from the early stage of 2DE, label-free, to SILAC and iTRAQ, which highly promotes our investigation on TCMs. Back to 1975, 2DE was first used to separate proteins [89] and it was generally applied in TCM research until 2004 [90]. As can be seen from Tables 1–3, the majority of proteomic approach for TCM studies is 2DE, which can only detect and quantify over 1000 proteins by comparing a couple of paired gels at one time [91]. For the inherited limitation of 2DE in protein separation [92], 2DE is gradually replaced by SILAC and iTRAQ, which can identify approximately 5000–8000 proteins in one experiment. In SILAC, an* in vivo* labeling approach that feeds cells with isotopically labeled “light” or “heavy” nutrients for different treatments, the altered proteins can be sensitively identified by MS/MS [90]. As an* in vitro* labeling method, iTRAQ can analyze up to eight different samples and thus is widely used in profiling animal tissues. According to PubMed as of April 2015, a total of 186 papers including 34 reviews related to the application of proteomics on TCMs are published. Among them, 17 recent reports involved TCMs characterization with advanced quantitative proteomics, revealing many more identifications of altered proteins for further mechanistic investigation. Obviously, advancements in systematic screening technology are required for profiling TCMs-induced protein alterations in depth, so that follow-up specific mechanistic researches become possible.

## 6. A New Dawn in TCM Investigation

As the rapid development in mass spectrometry, sophistic proteomics now has a capacity to identify over 10000 proteins at one time [93]. However, there still exist many limitations of mass spectrometry in protein identification, such as low identification rates for low-abundance proteins and poor-soluble proteins [94]. To deal with this problem, we recently introduced a new systematic profiling technique, translating mRNA analysis, or translatomic sequencing [95]. This method isolates ribosome-nascent chain-mRNA complex (RNC-mRNA) from cells/tissues and sequences the mRNAs released from the complex, and then the proteins corresponding to the translating mRNAs can be identified.

Under a steady state, the abundance of translating mRNAs corresponds to protein expressions [95], and thus phenotype-related protein alterations can be derived by comparing the RNC-mRNAs from two cellular conditions. For its independence from physical and chemical properties of proteins, translatomic sequencing has its inherent advantage in detecting translating mRNAs corresponding to the low-abundance and low-solubility proteins, thus offsetting the shortcoming of mass spectrometry. Translatomic sequencing has been successfully applied to identify “missing proteins” in Chromosome-Centric Human Proteome Project (C-HPP) [96]. We believe that, by integrating translatome sequencing with proteomics, systematic screening will bring TCM research to a new stage, where more comprehensive views about the TCM-induced biological alterations can be obtained and thus global investigations on the action mechanism of TCMs can be proceeded.

In view of the fact that mitochondria are the common target for most TCM compounds, a feasible strategy is to isolate mitochondria from the cells with and without treatments by TCMs, and then translatomics coupled with proteomics can be carried out to differentiate the mitochondrial mRNAs and proteins. This combination using both subcellular enrichment and in-depth transomics must provide comprehensive information for the identification of specific targets of TCMs in mitochondria. Similar ways can be applied to other subcellular fractionations; all together, the molecular mechanism of bioactive TCM components in cancer can be uncovered in a holistic view.

## Figures and Tables

**Table 1:** Summary of the application of proteomics to determine the mechanism of action of terpenoids.

Compounds	Proteomics method	Δ*ψ*_*m*_	ROS	Effects	Mechanism of action	Cell lines	References
*Sesquiterpene*							

*β*-Elemene	iTRAQ	N/A	N/A	N/A	Ribosome signaling, PPARs signaling, actin cytoskeleton, phagosome, biosynthesis, and amino acids metabolism	SGC7901	[23]

*β*-Elemene	N/A	↓	↑	Apoptosis↑	Release of cytochrome c/caspase-3	A549/DDP	[20]

*δ*-Elemene	N/A	↓	N/A	Apoptosis↑	p38 MAPK	NCI-H292	[97]

*β*-Elemene	N/A	↓	↑	Apoptosis↑	Release of cytochrome c/caspase-3/caspase-8/caspase-9	A2780/CP MCAS	[21]

*β*-Elemene	N/A	N/A	N/A	Apoptosis↑	Release of cytochrome c/caspase-3/caspase-8/caspase-9, p53	H23, H358, H460, and A549	[19]

Deoxyelephantopin	2-DE DIGE	N/A	N/A	Apoptosis↑	ER stress-independent apoptotic pathway	TS/A	[27]

Deoxyelephantopin	N/A	↓	↑	Apoptosis↑	Release of cytochrome c/caspase-3/caspase-7/caspase-8/caspase-10, Akt, ERK, and JNK pathways	CNE	[25]

Isodeoxyelephantopin	SILAC	↓	↑	Apoptosis↑	ROS-dependent DNA damage, mitochondrial-mediated apoptosis, and antitumor inflammation factor pathway	CNE1	[14]

Isodeoxyelephantopin	N/A	N/A	N/A	Apoptosis↑	NF-*κ*B	HL60, H1299, MM.1S, U266, and KBM-5	[82]

*Diterpene*							

Celastrol	SILAC	↓	↑	Apoptosis↑ Proliferation↓	Stress response, oxidative stress, and effects	Lymphoblastoid cells	[98]

Triptolide	2-DE	N/A	N/A	Proliferation↓	Perinuclear translocation of 14-3-3*ε*	SW480 and Lovo	[36]

Triptolide	N/A	↓	↑	Apoptosis↑	Release of cytochrome c/Smac/diablo, activation of caspase-3/caspase-9	HS-sultan, IM9, RPMI8226, and U266	[38]

Triptolide	N/A	↓	↑	Apoptosis↑	Release of cytochrome c, caspase-3/caspase-9, Bcl-2↓, Bax, and p53↑	L-02	[40]

Tanshinone IIA	2-DE	↓	↑	Apoptosis↑	p38, JNK/IRE1/PERK pathways, and ROS/ER stress pathways	CaSki	[99]

N/A: not applicable; ↑: upregulation; ↓: downregulation.

**Table 2:** Summary of the application of proteomics to determine the mechanism of action of flavonoids and glycosides.

Compounds	Proteomics methods	Δ*ψ*_*m*_	ROS	Effects	Mechanism of action	Cell lines	References
*Flavonoids*							

Baicalein	2-DE	N/A	↓	Proliferation↓	PRDX6↑ and ROS↓	DLD-1	[56]

Baicalein	N/A	↓	↑	Apoptosis↑	Mitochondrial respiration↑ and cytochrome c oxidase activity↑	H2.35	[57]

Luteolin	2-DE	N/A	N/A	Apoptosis↑	p38↓, HSP27↓, intracellular ATP levels↑, and mitochondrial activity↑	CH27	[100]

Quercetin	SILAC	N/A	N/A	Migration↓ Proliferation↓	IQGAP1 and *β*-tubulin	HepG2	[50]

Quercetin	2-DE	N/A	N/A	N/A	NADH dehydrogenase↓	SW480	[51]

Rotenone	SILAC	↓	↑	N/A	Oxygen-sensing pathway	SH-SY5Y	[101]

Panduratin A	iTRAQ	N/A	N/A	Angiogenesis↓	mTOR signaling↓	HUVECs	[102]

Calycosin	2-DE	N/A	N/A	Proliferation↓	Cell-cycling pathway	BEL-7402	[103]

Tea polyphenols	N/A	↓	↑	Apoptosis↑	NF-*κ*B	HeLa and SiHa	[104]

Tea polyphenols	N/A	↓	↑	Apoptosis↑	Bax and p53↑	Mouse skin cancer	[105]

*Glycosides*							

Ginsenoside Rb1	N/A	M	M	Apoptosis↓	Mitochondrial apoptotic pathway↓	NRC	[68]

Ginsenoside Rg1	2-DE	M	M	Apoptosis↓	eNOS pathway	HUVEC	[67]

Dioscin	N/A	↓	↑	Apoptosis↑	Mitochondria-initiated apoptosis pathway	HL-60	[18]

Dioscin	iTRAQ	↓	↑	Apoptosis↑	Oxidative phosphorylation, Wnt, p53, and calcium signaling pathways	HCT-116	[62]

M: maintenance; NRC: neonatal rat cardiomyocytes; HUVEC: human umbilical vein endothelial cells; N/A: not applicable; ↑: upregulation; ↓: downregulation.

**Table 3:** Summary of proteomics-based studies of paclitaxel resistance in various cancers.

Treatments	Proteomics methods	Cancer types	Cellular models	References
Paclitaxel-sensitive A549 versus paclitaxel-resistant A549	2-DE DIGE	Non-small-cell lung carcinoma	A549	[35]

Untreated versus paclitaxel versus MEK inhibitor versus paclitaxel + MEK inhibitor	2-DE	Non-small-cell lung carcinoma	H157	[30]

Untreated versus paclitaxel treated	2-DE DIGE	Breast cancer	MDA-MB-435S	[106]

Untreated versus paclitaxel treated	2-DE	Cervical cancer	Hela	[107]

Untreated versus paclitaxel treated	2-DE DIGE	Promyeloid leukaemia	HL-60	[108]

Untreated versus paclitaxel treated	2-DE	Dermal papilla	Dermal papilla	[109]

Paclitaxel-sensitive CaOV3 versus paclitaxel-resistant variant CaOV3	N/A	Ovarian cancer	CaOV3 and OV90	[32]

SK-BR-3 versus paclitaxel-resistant SK-BR-3	2-DE	Breast cancer	SK-BR-3	[34]

N/A: not applicable.
